# Photobiomodulation at 660 nm enhances proliferative activity while preserving viability in human endothelial cells in vitro

**DOI:** 10.1007/s10561-026-10237-z

**Published:** 2026-07-22

**Authors:** Diego Filgueira Albuquerque, Vladimir Galdino Sabino, Naisandra Bezerra da Silva Farias, Pedro Paulo de Andrade Santos, Manoela Domingues Martins, Janine Karla França da Silva Braz, Carlos Augusto Galvão Barboza

**Affiliations:** 1 Postgraduate Program in Structural and Functional Biology, Federal University of Rio Grande do Norte, Natal, Brazil; 2 Postgraduate Program in Biochemistry and Molecular Biology, Federal University of Rio Grande do Norte, Natal, Brazil; 3 Department of Morphology, Federal University of Rio Grande do Norte, Natal, Brazil; 4 Department of Oral Pathology, Federal University of Rio Grande do Sul, Porto Alegre, Brazil; 5 National Institute of Science and Technology in Translational Biophotonics in Dentistry, Porto Alegre, Brazil

**Keywords:** Photobiomodulation therapy, Endothelial cells, Cell proliferation, Cell survival, Tissue engineering

## Abstract

Efficient endothelialization is essential for vascular graft integration and long-term performance, yet strategies to enhance endothelial proliferative activity remain limited. Photobiomodulation (PBM) is a promising noninvasive approach for modulating cell behavior, although its effects on endothelial cells under standard culture conditions remain insufficiently defined. This in this study evaluated the effects of 660 nm PBM at different energy densities on the proliferative activity and viability of human umbilical vein endothelial cells cultured under nonstress conditions. Cells were assigned to four groups: nonirradiated control and PBM-treated groups exposed to 1.0, 4.0, or 7.5 J/cm^2^. Metabolic/proliferative activity and viability were assessed at 24, 48, and 72 h using Alamar Blue, Live/Dead staining, Annexin V/propidium iodide labeling, cell cycle analysis, and Ki67 fluorescence intensity. At 72 h, all irradiated groups showed significantly greater metabolic activity than the control group (1.0 J/cm^2^, *p* < 0.01; 4.0 and 7.5 J/cm^2^, *p* < 0.001), whereas viability remained high across all groups (> 95%), with no evidence of cytotoxicity. PBM redistributed cells toward proliferative phases, with a higher proportion of S-phase cells at 1.0 J/cm^2^ and greater accumulation in G2/M at 4.0 and 7.5 J/cm^2^. Ki67 fluorescence intensity also increased and was highest at 7.5 J/cm^2^ (*p* < 0.05). Overall, 660 nm PBM enhanced endothelial proliferative activity while preserving cell viability, with coordinated changes in cell cycle progression and Ki67 fluorescence intensity across different energy densities and the most pronounced overall response at 7.5 J/cm^2^. These findings support PBM as a promising adjunctive strategy for modulating endothelial behavior in vascular tissue engineering and highlight its relevance under standard nonstress culture conditions.

**Supplementary Information:**

The online version contains supplementary material available at https://doi.org/10.1007/s10561-026-10237-z.

## Introduction

In recent decades, tissue engineering has advanced considerably toward the development of functional substitutes for damaged tissues and organs. However, the generation of a stable, hierarchical vascular network remains one of the major obstacles to the successful fabrication of large and complex constructs, which depend on an efficient supply of oxygen and nutrients (Margolis et al. 2023). The establishment of functional microvascular networks within engineered tissues is therefore essential to ensure cell survival, tissue integration, and long-term construct performance after implantation. In this context, in vitro prevascularization strategies have been developed to promote the formation of capillary-like networks prior to implantation, thereby improving vascular connectivity and graft performance in vivo (Rademakers et al. 2019).

Endothelial cells (ECs) are central to these processes, as they form the inner lining of blood vessels and regulate vascular homeostasis, angiogenesis, and extracellular matrix remodeling. The establishment of a functional endothelial layer, known as endothelialization, is critical for preventing thrombosis, promoting vascular integration, and supporting long-term graft functionality (Soletti et al. 2010; Ahmed et al. 2011; Seifu et al. 2013; Wu et al. 2018). Accordingly, successful vascular constructs must provide a microenvironment capable of supporting EC adhesion, spreading, proliferation, and extracellular matrix production (Ahmed et al. 2011; Wu et al. 2018).

Several strategies have been proposed to enhance endothelialization, including biomaterial surface modification, the incorporation of angiogenic growth factors, and the recruitment or differentiation of endothelial progenitor cells (Simunovic and Finkenzeller 2021; Rosellini et al. 2022). Although these approaches have yielded promising results, they are often associated with greater system complexity, higher costs, and limited control over cellular responses. In addition, obtaining a sufficient number of viable endothelial cells with robust proliferative potential remains challenging, as cellular behavior is strongly influenced by donor-related factors and culture conditions (Devillard et al. 2021). In this context, standardized cell sources, including banked endothelial cell preparations or experimentally established endothelial cell lines, may support reproducibility and scalability in regenerative strategies. Therefore, approaches capable of functionally modulating endothelial cells before their therapeutic or bioengineering use may also be relevant from a broader cell-banking perspective.

Photobiomodulation (PBM) via low-level laser irradiation has emerged as a noninvasive strategy for modulating cellular behavior and enhancing proliferation in different cell types (Góralczyk et al. 2015; de Freitas and Hamblin 2016; Ginani et al. 2018; da Silva et al. 2023). PBM has been proposed as a complementary tool in tissue engineering, potentially acting alongside the classical triad of cells, scaffolds, and growth factors (Marques et al. 2016). In endothelial cells, PBM has been associated with increased proliferation, migration, cytoskeletal organization, and expression of angiogenic mediators such as vascular endothelial growth factor (VEGF) (Schindl et al. 2003; Ricci et al. 2009; Silva et al. 2010; Szymanska et al. 2013; Góralczyk et al. 2015; Li et al. 2020). Representative endothelial studies have reported proliferative responses to red-light PBM in the 635–670 nm range, including 670 nm at 2–8 J/cm^2^ (Schindl et al. 2003) and 635 nm at 2, 4, and 8 J/cm^2^ (Szymanska et al. 2013; Góralczyk et al. 2015). More recent HUVEC studies have also shown parameter-dependent responses under low-dose regimens in the 1–4 J/cm^2^ range and under broader comparisons extending to 20 J/cm^2^ and different wavelengths, reinforcing that endothelial PBM effects depend on the combined interaction of spectral and dosimetric conditions (Li et al. 2020; Terena et al. 2021). Moreover, broader wavelength comparisons have shown that endothelial responses are not determined by red light alone but by the combined interaction between wavelength and dosimetric conditions, including irradiation regimens extending into the infrared range (Terena et al. 2021; Joniová et al. 2024).

PBM has also been reported to increase cell viability, protect endothelial cells from stress-induced damage (Walter et al. 2015; Chu et al. 2018; Terena et al. 2021), and promote angiogenesis in vivo (Cury et al. 2013). Recent studies further indicate that PBM modulates endothelial responses under pathological conditions, including ischemic and metabolic stress (da Silva Oliveira et al. 2025). Moreover, PBM has been associated with enhanced mitochondrial function, increased nitric oxide bioavailability, and activation of angiogenic signaling pathways in endothelial cells (Amaroli et al. 2019; Yokomizo et al. 2022; Joniová et al. 2024), reinforcing its relevance as a regulator of vascular cell behavior.

Despite these promising findings, the biological effects of PBM on endothelial cells are highly dependent on irradiation parameters, including wavelength, energy density, and exposure time. Considerable variability among studies has hindered the definition of optimal protocols, particularly with respect to dose-related responses and the balance between biostimulatory effects and potential cytotoxicity (Peplow et al. 2010; Szymanska et al. 2013; Góralczyk et al. 2015; Chu et al. 2018; Li et al. 2020). Moreover, most available studies have focused on stress or pathological models, whereas the effects of PBM under standard physiological conditions relevant to tissue engineering remain insufficiently explored. This gap limits both protocol standardization and the broader translation of PBM into reproducible tissue engineering strategies.

Available evidence suggests that endothelial responses to PBM are determined by the combined interaction of spectral and dosimetric parameters rather than by isolated variables, further highlighting the complexity of protocol optimization (Rohringer et al. 2017; Joniová et al. 2024). In this context, comparative evaluation of low, intermediate, and high energy densities within ranges previously reported to influence endothelial cell behavior may contribute to a more rational understanding of dose-dependent endothelial PBM responses (Szymanska et al. 2013; Góralczyk et al. 2015; Li et al. 2020; Terena et al. 2021). Therefore, a clearer understanding of how different PBM parameters affect endothelial proliferation and viability is essential for advancing the rational application of PBM in tissue engineering.

Given these considerations, the present study aimed to evaluate the effects of 660 nm photobiomodulation at different energy densities (1.0, 4.0, and 7.5 J/cm^2^) on the proliferative activity and viability of human umbilical vein endothelial cells (HUVECs) cultured under standard conditions without the induction of cellular stress. We hypothesized that 660-nm PBM modulates endothelial proliferative behavior under standard culture conditions by enhancing proliferative activity, preserving viability, and inducing coordinated changes in cell cycle distribution and Ki67-related proliferative signaling across different energy densities, thereby supporting its potential role in endothelialization-related processes relevant to tissue engineering.

## Materials and methods

### Cell culture

Human umbilical vein endothelial cells (HUVECs-C; ATCC^®^ CRL-1730™; ATCC, USA) were cultured in Dulbecco’s modified Eagle’s medium (DMEM) supplemented with 10% fetal bovine serum (FBS) and 1% antibiotic–antimycotic solution (Gibco, USA). The cells were maintained in a humidified incubator (Thermo Fisher Scientific, USA) at 37 °C and 5% CO₂. All experiments were performed using HUVECs at passages 4–5 (P4–P5).

### Study design

**Fig. 1:**
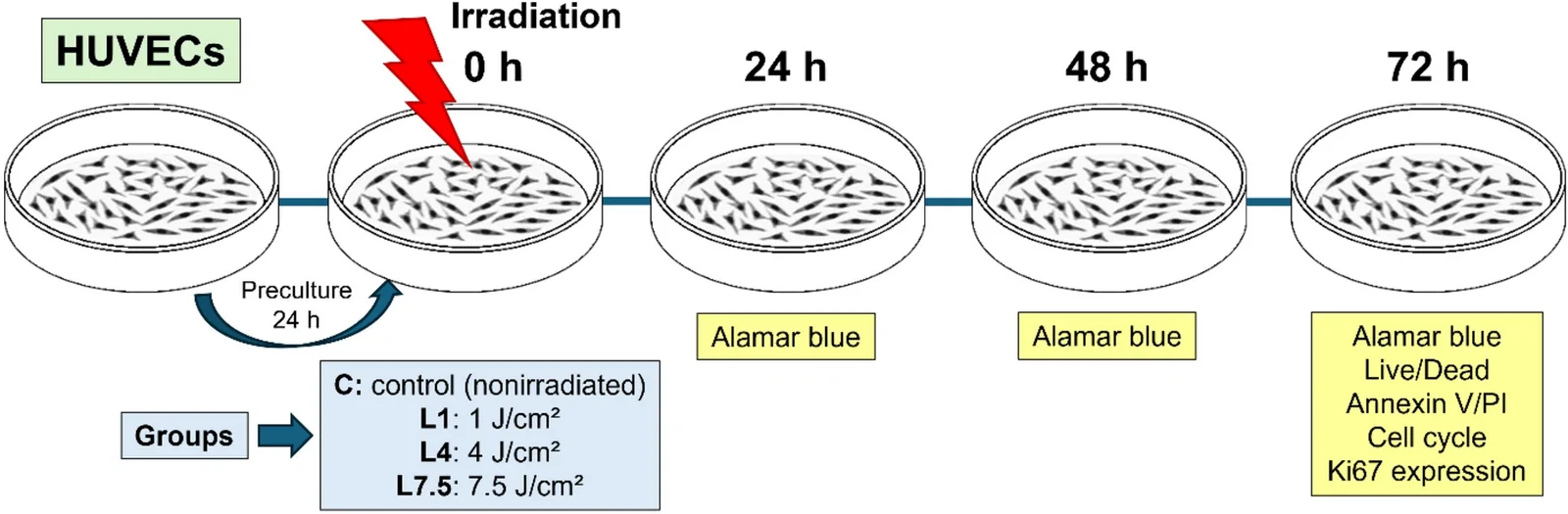
Schematic representation of the experimental design. HUVECs were precultured for 24 h to allow cell attachment, irradiated at 0 h, and evaluated after 24, 48, and 72 h. The experimental groups were C (nonirradiated control), L1 (1.0 J/cm^2^), L4 (4.0 J/cm^2^), and L7.5 (7.5 J/cm^2^). The Alamar Blue assay was performed at all time points, whereas Live/Dead staining, Annexin V/propidium iodide (PI) labeling, cell cycle analysis, and Ki67 expression analysis were performed at 72 h

After 24 h of preculture on the polystyrene surfaces of culture plates (TTP, Switzerland) to allow cell attachment, HUVECs were assigned to four groups: C (control), nonirradiated cells maintained under standard culture conditions; L1, cells irradiated at 1.0 J/cm^2^; L4, cells irradiated at 4.0 J/cm^2^; and L7.5, cells irradiated at 7.5 J/cm^2^. The cells were analyzed at 24, 48, and 72 h after irradiation using biochemical and morphological assays to assess their viability and proliferative activity (Fig. 1). All experiments were performed in quadruplicate. Each multiwell culture plate contained samples from the different experimental groups, allowing irradiated and nonirradiated cells to remain under equivalent environmental conditions during the irradiation procedure. The assays were selected to provide complementary information on metabolic activity, membrane integrity, apoptosis, and proliferation status.

### Photobiomodulation protocol

Irradiation was performed using an InGaAlP diode laser device (Kondortech, Brazil) operating at a wavelength of 660 nm, with an output power of 30 mW, a fiber tip diameter of 0.12 cm, a nominal beam spot area of 0.0113 cm^2^, a round beam profile, and continuous emission mode. The output power of the laser was measured with a LaserCheck Power Meter (Coherent Inc., USA) before each application. The applied radiant exposures were nominally set at 1.0, 4.0, and 7.5 J/cm^2^, corresponding to irradiation times of 33.3, 133.3, and 250.0 s per application point, respectively. Because different plate formats were used depending on the assay, the irradiation geometry was adapted to each experimental configuration (Table 1). Accordingly, these nominal settings should be interpreted in light of the assay-specific application setup rather than as strictly equivalent whole-well dosimetric conditions across all assays. To minimize unintended light scattering between wells during irradiation, HUVECs were seeded in culture plates with empty wells interspersed between the experimental wells. The irradiation probe was positioned perpendicular to the plate at a fixed distance of 0.5 cm from the cell monolayer using a standardized holder. For the assays performed in 6-well plates, irradiation was applied at five equidistant points to provide a more homogeneous spatial distribution of light over the culture area, with the same irradiation time applied at each point. All irradiations were performed once at T0 after the 24 h preculture period.

**Table 1:** Assay-specific photobiomodulation application conditions used in the study

Parameter	Alamar Blue assay	Live/Dead, Annexin V/PI, Ki67, and cell cycle assays
Plate format	96-well plate	6-well plate
Number of irradiation points per sample	1	5
Irradiation pattern	Central irradiation	Fixed equidistant points
Nominal radiant exposure (J/cm^2^)	1.0, 4.0, and 7.5	1.0, 4.0, and 7.5
Irradiation time per point (s)	33.3, 133.3, and 250.0	33.3, 133.3, and 250.0
Irradiation timing	Single irradiation at T0	Single irradiation at T0
Analysis time points	24, 48, and 72 h	72 h

T0 indicates the time of laser application after the 24 h preculture period. The same wavelength, output power, nominal radiant exposure, and irradiation time per point were used in all the assays, whereas the irradiation geometry was adapted according to the plate format and experimental configuration. For the 6-well assays, the total irradiation time per sample corresponded to the sum of the five equidistant irradiation points

### Cell proliferation assay

Cell proliferation was evaluated at 24, 48, and 72 h after irradiation using the Alamar Blue assay (Invitrogen, USA). This fluorometric method assesses cellular metabolic activity based on the reduction of resazurin (7-hydroxy-3H-phenoxazine-3-one 10-oxide) to resorufin. The assay was performed at all time points to monitor temporal changes in cellular metabolic activity after irradiation.

Cells were seeded in 96-well plates at a density of 5 × 10^3^ cells per well and subjected to the photobiomodulation protocol, except for the control group. At each experimental time point, the culture medium was removed, and the cells were incubated with 10 µL of Alamar Blue reagent and 90 µL of Dulbecco’s modified Eagle’s medium (DMEM; Gibco, USA) for 4 h at 37 °C in a humidified atmosphere containing 5% CO₂. The absorbance was then measured using a microplate reader (Epoch, Biotek Instruments, USA) at 570 and 600 nm, corresponding to the reduced and oxidized forms of the reagent, respectively. The percentage reduction of Alamar Blue reagent was calculated according to the manufacturer’s equation based on the molar extinction coefficients of the oxidized and reduced forms of the reagent (Supplementary Material 1).

### Cell viability assay

Cell viability was assessed using the Live/Dead^®^ Viability/Cytotoxicity Kit for Mammalian Cells (Invitrogen, USA). Cells were seeded in 6-well plates at a density of 2 × 10^5^ cells per well. At 72 h after irradiation, the cells were washed with phosphate-buffered saline (PBS) and incubated with 2 µM calcein AM and 4 µM ethidium homodimer-1 for 20 min at room temperature in the dark. The samples were examined under a fluorescence microscope (Zeiss Imager A2, Oberkochen, Germany), and photomicrographs (n = 4) of the central field of each sample were obtained at 100 × magnification. The images were analyzed using ImageJ software to count viable (green) and dead (red) cells. For comparisons among groups, the absolute number of viable cells and the percentage of dead cells were considered, and the latter was calculated as follows: % dead cells = number of dead cells/total number of cells × 100.

### Cell death assay

Cell death was assessed by flow cytometry using a FITC Annexin V/Dead Cell Apoptosis Kit (Invitrogen, USA). Cells were seeded in 6-well plates at a density of 2 × 10^5^ cells per well. At 72 h after irradiation, the cells were washed with PBS, resuspended in 1 × binding buffer, and incubated for 15 min at room temperature in the dark with 5 µL of Annexin V–FITC and 1 µL of propidium iodide (PI). Annexin V–FITC was used to identify early apoptotic cells, whereas PI was used to detect late apoptotic and necrotic cells. A total of 10,000 events per sample were acquired using a FACSCanto II flow cytometer (BD Biosciences, USA).

### Cell cycle analysis

The cell cycle distribution was analyzed by flow cytometry using propidium iodide (PI) staining. HUVECs were seeded in 6-well plates at a density of 2 × 10^5^ cells per well and cultured for 72 h after irradiation. The cells were then detached from the plates by trypsinization, collected, washed with PBS, fixed in cold 70% ethanol, and stored at 4 °C until analysis. Before acquisition, the cells were washed and resuspended in PBS containing propidium iodide (PI) and RNase A to ensure specific DNA staining. Samples were analyzed using a FACSCanto II flow cytometer (BD Biosciences, USA), and 10,000 events were acquired per sample. Cell populations corresponding to the G0/G1, S, and G2/M phases were determined from DNA content histograms using FlowJo v10 software (BD Biosciences, USA), following standard gating strategies to exclude debris and cell doublets.

### Ki67 expression analysis

Ki67 expression was evaluated by flow cytometry. HUVECs were seeded in 6-well plates at a density of 2 × 10^5^ cells per well and analyzed 72 h after irradiation. The cells were then detached from the plates by trypsinization, collected, washed with PBS, fixed and permeabilized according to standard protocols, and incubated with 10 µL of FITC-conjugated anti-Ki67 monoclonal antibody (clone 7B11; Invitrogen, USA) for 20 min at room temperature in the dark. After incubation, the samples were analyzed using a FACSCanto II flow cytometer (BD Biosciences, USA), and 10,000 events were acquired per sample. The mean fluorescence intensity (MFI) of Ki67 was determined using FlowJo v10 software (BD Biosciences, USA) as a quantitative estimate of relative protein expression across the experimental groups.

### Statistical analysis

Quantitative data, including the percentage of Alamar Blue reduction, the number of viable cells per field, the percentage of dead cells in the Live/Dead assay, and the mean fluorescence intensity (MFI) of Ki67, were initially assessed for normality using the Shapiro–Wilk test in GraphPad Prism 8 (GraphPad Software, USA). Given that the data did not follow a normal distribution, nonparametric tests were applied. Differences among groups at each time point were analyzed using the Kruskal–Wallis test, followed by Dunn’s multiple comparisons test. Statistical significance was set at *p* < 0.05.

## Results

### Cell proliferation assay

**Fig. 2:**
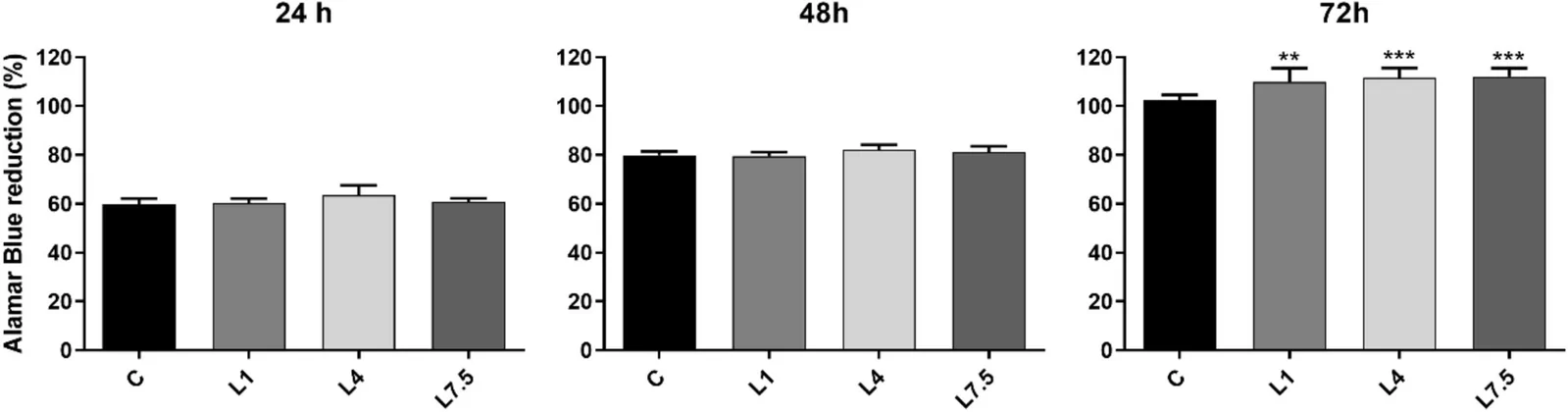
Percentage of Alamar Blue reduction (mean ± standard deviation) in each group at 24, 48, and 72 h. Asterisks indicate statistically significant differences compared with the control group (**p < 0.01; ***p < 0.001; Kruskal–Wallis test followed by Dunn’s multiple comparisons test)

Assessment of cellular metabolic activity by the Alamar Blue assay revealed no significant differences between the irradiated and control groups at 24 or 48 h. In contrast, at 72 h, compared with the control group, all the irradiated groups showed significantly greater metabolic activity, with the L4 and L7.5 groups exhibiting the most pronounced increases (*p* < 0.001) (Fig. 2).

### Cell viability assay

**Fig. 3:**
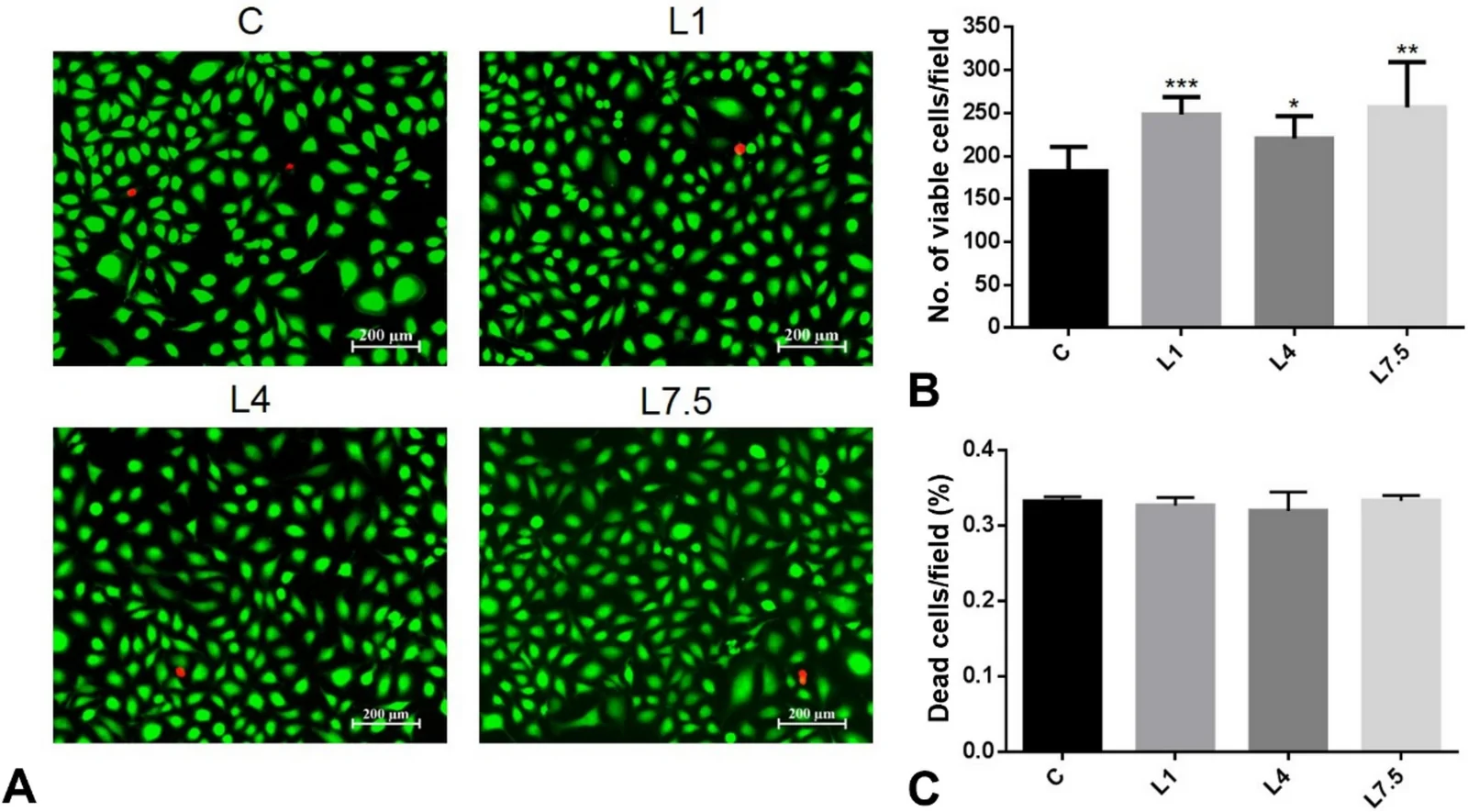
**A** Representative fluorescence micrographs of HUVECs at 72 h showing viable cells stained with calcein AM (green) and nonviable cells stained with ethidium homodimer-1 (red). Scale bar = 200 µm. **B** Number of viable cells per field in the different groups. Asterisks indicate statistically significant differences compared with the control group (*p < 0.05; **p < 0.01; ***p < 0.001; Kruskal–Wallis test followed by Dunn’s multiple comparisons test). **C** Percentage of dead cells per field in the different groups, with no statistically significant differences among them (*p* > 0.05; Kruskal–Wallis test followed by Dunn’s multiple comparisons test)

Representative fluorescence micrographs obtained at 72 h revealed a predominance of viable cells stained with calcein AM (green) and only sparse nonviable cells stained with ethidium homodimer-1 (red) in all groups (Fig. 3A). Quantitative analysis revealed significantly greater numbers of viable cells per field in all the irradiated groups than in the control group, with the most pronounced increases observed in the L1 and L7.5 groups (Fig. 3B). Although slight differences in the percentage of dead cells per field were observed, no statistically significant differences were detected among the groups (Fig. 3C). These findings are consistent with the Alamar Blue assay results and indicate that PBM increased cell density while maintaining high viability.

### Cell death assay

**Fig. 4:**
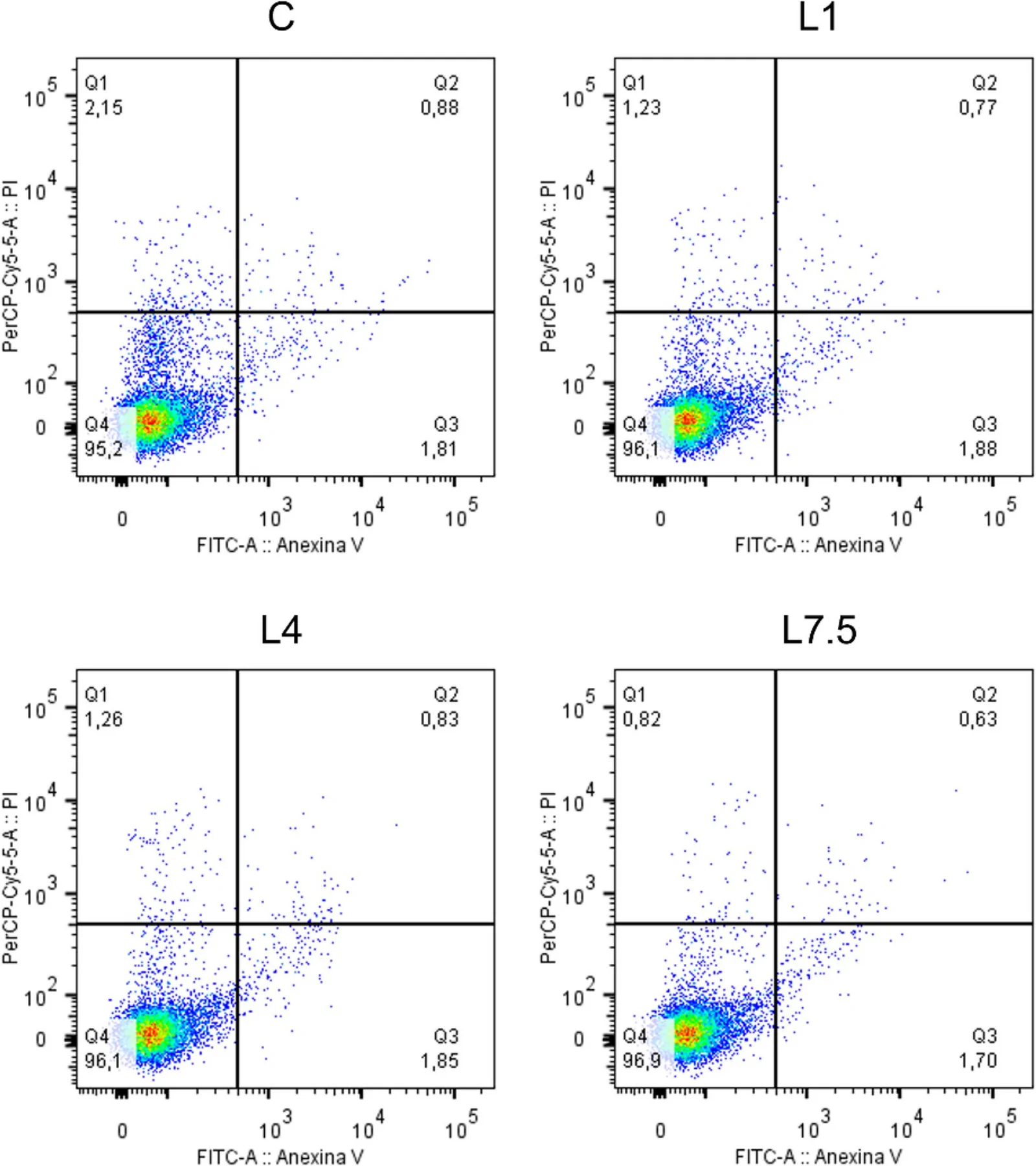
Representative flow cytometry dot plots of Annexin V–FITC/PI staining in HUVECs at 72 h. Q1, Annexin V⁻/PI⁺ (necrotic cells); Q2, Annexin V⁺/PI⁺ (late apoptotic cells); Q3, Annexin V⁺/PI⁻ (early apoptotic cells); and Q4, Annexin V⁻/PI⁻ (viable cells)

Representative Annexin V/PI flow cytometry dot plots obtained at 72 h revealed a high percentage of viable cells (Annexin V⁻/PI⁻) in all groups (Fig. 4). The percentage of viable cells remained consistently high, ranging from 95.2% in the control group to 96.9% in the L7.5 group. No evident increase in early apoptotic (Annexin V⁺/PI⁻), late apoptotic (Annexin V⁺/PI⁺), or necrotic (Annexin V⁻/PI⁺) cell populations was observed in the irradiated groups. These findings are consistent with the results of the Live/Dead and Alamar Blue assays, indicating that PBM did not induce detectable cytotoxic effects under the experimental conditions.

### Cell cycle analysis

**Fig. 5:**
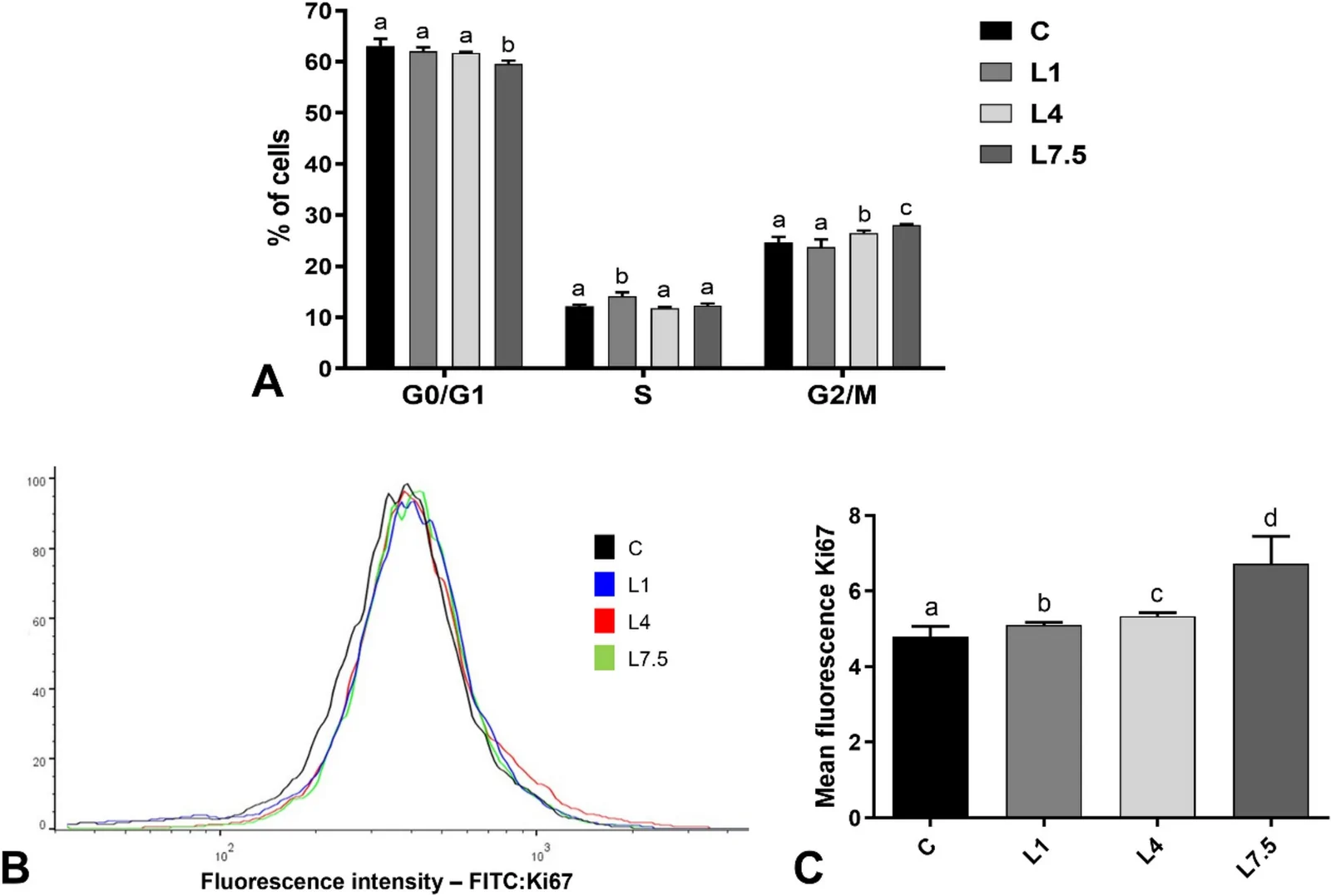
**A** Percentage of cells (mean ± standard deviation) in the G0/G1, S, and G2/M phases at 72 h in the different experimental groups. Different lowercase letters indicate statistically significant differences among groups within each cell cycle phase (p < 0.05; Kruskal–Wallis test followed by Dunn’s multiple comparisons test). **B** Representative flow cytometry histograms of Ki67 fluorescence intensity in HUVECs at 72 h. **C** The mean fluorescence intensity (MFI) of Ki67 in the experimental groups. Different lowercase letters indicate statistically significant differences among groups (*p* < 0.05; Kruskal–Wallis test followed by Dunn’s multiple comparisons test)

Cell cycle analysis at 72 h revealed a redistribution of the cell population across proliferative phases in the irradiated groups (Fig. 5A). Compared with the other groups, the L7.5 group exhibited a significantly lower percentage of cells in G0/G1 (*p* < 0.05), indicating reduced retention in the resting/initial growth phase. In the S phase, compared with the remaining groups, only the L1 group contained a significantly greater percentage of cells (*p* < 0.05). In contrast, the proportion of cells in G2/M increased with increasing irradiation dose, with significantly greater values in the L4 and L7.5 groups than in the C and L1 groups, and the highest percentage was observed in the L7.5 group (*p* < 0.05). Together, these findings indicate that PBM modulates cell cycle progression, with higher energy densities favoring accumulation in the G2/M phase.

### Ki67 expression

Flow cytometry analysis revealed greater Ki67 fluorescence intensity in all the irradiated groups than in the control group, with a progressive increase across doses and the highest value observed in the L7.5 group (Fig. 5B, C). These findings are consistent with the cell cycle results and support the proliferative effect of PBM on HUVECs.

## Discussion

Photobiomodulation (PBM) has been investigated for its biostimulatory effects for several decades; however, defining optimal irradiation protocols for specific cell types remains a major challenge (de Freitas and Hamblin 2016; da Silva et al. 2023). In vascular tissue engineering, this issue is particularly relevant because endothelial cells play a central role in endothelialization, angiogenesis, and graft integration. The results of the present study demonstrated that the 660 nm PBM enhances HUVEC proliferative activity while maintaining high cell viability, with the most pronounced overall response observed at 7.5 J/cm^2^. These findings are consistent with a growing body of in vitro evidence indicating that PBM positively modulates endothelial cell behavior (Winter et al. 2018; Joniová et al. 2024). Importantly, the results of the present study extend this evidence by showing that under nonstress culture conditions, the 660 nm PBM modulates endothelial behavior at multiple complementary levels, including metabolic/proliferative activity, viability, cell cycle progression, and Ki67-associated proliferative signaling across different energy densities, rather than merely increasing cellular metabolic output. This interpretation is further supported by recent review evidence indicating that PBM is a noninvasive regenerative tool that can enhance tissue engineering outcomes both as a standalone intervention and in combination with the classical triad of cells, scaffolds, and signaling molecules (Selestin Raja et al. 2024).

The experiments were conducted using HUVECs maintained under standard culture conditions, which approximate those commonly adopted in vascular tissue engineering models. Their well-established ability to form robust microvascular networks both in vitro and in vivo supports their use as a reliable endothelial model (Margolis et al. 2023). Unlike other cell types, in which PBM effects may be enhanced under nutritional stress (Tagliani et al. 2010; Moura-Netto et al. 2016; da Silva et al. 2019), endothelial responses appear to be strongly context dependent and influenced by metabolic status, oxygen availability, and irradiation parameters (Malta et al. 2021). Nevertheless, PBM has demonstrated positive effects not only under physiological conditions (Terena et al. 2021) but also in adverse environments such as hyperglycemic and ischemic conditions (Góralczyk et al. 2016; Feng et al. 2024; da Silva Oliveira et al. 2025), particularly in metabolically compromised settings, reinforcing its potential relevance for vascular regenerative applications.

The selection of a 660 nm wavelength was based on consistent evidence supporting the efficacy of visible red light in promoting endothelial proliferation. Previous studies have demonstrated increased HUVEC proliferation with 670 nm irradiation at 2–8 J/cm^2^ (Schindl et al. 2003) and with 635 nm irradiation at 2, 4, and 8 J/cm^2^ (Szymanska et al. 2013; Góralczyk et al. 2015), findings further supported by reports showing enhanced proliferation, migration, and cytoskeletal organization under related red-light conditions (Oh et al. 2018; Winter et al. 2018). More recent studies have also shown that endothelial responses remain favorable within low-dose regimens in the 1–4 J/cm^2^ range (Li et al. 2020), whereas broader comparisons using 660 and 780 nm at 1, 5, 10, and 20 J/cm^2^ have highlighted the importance of wavelength-dependent effects on HUVEC behavior (Terena et al. 2021). In addition, an optimization study comparing 652, 689, 730, and 808 nm at irradiances ranging from 1.5 to 25 mW/cm^2^ revealed that endothelial outcomes depend on the combined interaction among wavelength, irradiance, and total delivered energy (Joniová et al. 2024). However, endothelial responses are not determined by wavelength alone. Infrared irradiation, for example, has been associated with reduced viability under certain conditions (Terena et al. 2021), and high fluences can induce cellular stress or death (Rohringer et al. 2017). Together, these findings indicate that PBM outcomes depend on the interaction between wavelength and dosimetric parameters, with the potential to shift from stimulation to stress depending on irradiation conditions. Accordingly, wavelength should be interpreted within a broader dosimetric framework, although red light remains among the most frequently reported effective ranges for endothelial proliferation in vitro (Schindl et al. 2003; Góralczyk et al. 2015; Li et al. 2020; Terena et al. 2021).

PBM at 1.0, 4.0, and 7.5 J/cm^2^ increased HUVEC proliferative activity, particularly at 72 h, in agreement with previously reported effective fluences. For example, red-light PBM at 2–8 J/cm^2^ has been associated with endothelial proliferation (Schindl et al. 2003), whereas other red-light regimens at 2, 4, and 8 J/cm^2^ also promoted favorable endothelial responses (Szymanska et al. 2013; Góralczyk et al. 2015). Temporal dynamics also appear to play an important role, as increased proliferation has been reported from the second day onward across 1–20 J/cm^2^ depending on wavelength and experimental configuration (Terena et al. 2021), and PBM-induced effects have been shown to be both dose- and time-dependent under low-dose regimens in the 1–4 J/cm^2^ range (Li et al. 2020). More recent evidence indicates that endothelial responses result from multidimensional interactions among wavelength, irradiance, and total delivered energy rather than from a single parameter. In this context, Joniová et al. systematically evaluated endothelial responses under 652, 689, 730, and 808 nm illumination combined with irradiances from 1.5 to 25 mW/cm^2^, demonstrating that angiogenic and mitochondrial outcomes depend on the combined radiometric and spectral configuration (Joniová et al. 2024). In this context, the enhanced proliferative response observed at 7.5 J/cm^2^ without loss of viability suggests that this dose falls within an effective range for endothelial stimulation under the present experimental conditions. This interpretation is consistent with the biphasic dose–response model (Schindl et al. 2003; Feng et al. 2012; Li et al. 2020), in which insufficient doses produce limited effects and excessive doses may become inhibitory or cytotoxic (Du et al. 2012; Bagheri et al. 2018). This view is consistent with a recent comprehensive review showing that PBM outcomes are governed by the combined interaction of wavelength, irradiance, energy density, irradiation time, and treatment interval and that both insufficient and excessive doses may limit the biological response (Selestin Raja et al. 2024).

Although the present study did not directly assess mitochondrial activity, reactive oxygen species generation, nitric oxide bioavailability, or proangiogenic signaling pathways, the coordinated increase in proliferative activity, cell cycle progression, and Ki67 fluorescence intensity observed here is biologically consistent with mechanisms previously proposed for endothelial photobiomodulation. Previous studies have associated PBM with cytochrome c oxidase–related mitochondrial activation and oxidative phosphorylation in endothelial cells, as well as with nitric oxide modulation and angiogenic pathway regulation in endothelial models (Karu 2010; Amaroli et al. 2019; Li et al. 2020; Yokomizo et al. 2022; da Silva Oliveira et al. 2025). Accordingly, these pathways should be interpreted here as plausible explanatory hypotheses rather than as mechanisms directly demonstrated by the present data.

With respect to cell cycle dynamics, PBM promoted a redistribution of cells toward the proliferative phases. The reduction in the proportion of cells in the G0/G1 phase, particularly in the L7.5 group, indicates enhanced cell cycle progression rather than simple preservation of cell viability. This shift is consistent with previous reports showing that PBM favors entry into DNA synthesis and mitosis (Feng et al. 2012). At the lower energy density of 1.0 J/cm^2^, a higher proportion of cells was observed in the S phase, suggesting enhanced entry into DNA synthesis. In contrast, higher energy densities (4.0 and 7.5 J/cm^2^) were associated with a greater proportion of cells in the G2/M phase, indicating progression toward mitosis. This pattern suggests that PBM modulates cell cycle progression through phase-specific effects that vary according to energy density.

The increase in Ki67 fluorescence intensity, particularly at higher doses, further supports these findings. Because Ki67 is expressed throughout all active phases of the cell cycle but is absent in quiescent cells, the greater fluorescence intensity observed in the irradiated groups corroborates the observed shift toward proliferative states. These results are consistent with both the cell cycle data and previous studies reporting enhanced endothelial proliferation following PBM (Feng et al. 2012; Winter et al. 2018; Li et al. 2020; Joniová et al. 2024). Taken together, these findings suggest that PBM enhances endothelial proliferative activity not only by increasing metabolic activity but also by supporting coordinated progression through the cell cycle.

Importantly, cell viability remained high across all groups, with no evidence of cytotoxicity under the tested conditions. This dual effect is supported by studies demonstrating PBM-mediated cytoprotection through the inhibition of inflammatory pathways such as p38 MAPK and NF-κB (Chu et al. 2018), as well as the modulation of oxidative stress and endothelial dysfunction under ischemic conditions (Feng et al. 2024). Taken together, these findings are consistent with the absence of detectable cytotoxic effects under the experimental conditions used here.

From a translational perspective, these results are relevant because successful endothelialization depends on rapid endothelial coverage capable of preventing thrombosis and supporting vascular remodeling (Wu et al. 2018; Devillard et al. 2021; Rosellini et al. 2022). Although VEGF- and other growth factor-based strategies have shown angiogenic potential, their application may be limited by a short biological half-life, rapid diffusion from the delivery site, dose-control challenges, and the complexity of achieving controlled exogenous delivery, which may compromise cost-effectiveness (Ren et al. 2020; Beheshtizadeh et al. 2023). In contrast, PBM offers a noninvasive strategy for enhancing endothelial performance without the need for exogenous growth factors or complex biomaterial modifications. In addition to promoting proliferation, PBM has been associated with increased migration, angiogenic signaling, and cytoskeletal organization (Cury et al. 2013; da Silva Oliveira et al. 2025), as well as with effects relevant to cell–biomaterial interactions, including adhesion and matrix deposition. These properties position PBM as a complementary tool within the tissue engineering framework, potentially acting alongside the classical triad of cells, scaffolds, and signaling molecules. Beyond scaffold-based applications, these findings may also be relevant in contexts in which endothelial cells are expanded, standardized, or obtained from banked sources before experimental or translational use, as PBM may serve as a noninvasive strategy for preapplication functional modulation.

The present study has some limitations. The use of two-dimensional cultures does not fully reproduce the complexity of three-dimensional vascular environments, and the 72 h observation period may not capture longer-term effects such as network formation or phenotypic stabilization. Only one wavelength and a single-irradiation protocol were evaluated, and no functional angiogenic assays, such as migration or capillary-like network formation, were performed. In addition, because endothelial cell behavior may vary according to passage history and replicative status, these functional assays should ideally be incorporated into a fully integrated experimental design rather than added in isolation under nonequivalent experimental conditions. Moreover, the molecular mechanisms underlying the observed effects were not directly investigated and therefore remain supported by literature-based hypotheses rather than by direct experimental evidence from the present study. Future studies should therefore explore PBM effects in three-dimensional systems, such as endothelialized scaffolds or hydrogel-based cultures, coculture models involving endothelial cells and stromal support cells, longer experimental time frames beyond 72 h to assess sustained proliferative behavior and phenotypic stabilization, and functional endothelial assays such as migration and capillary-like network formation to better approximate clinically relevant conditions.

In summary, PBM at 660 nm enhances HUVEC proliferative activity while maintaining high cell viability. Among the tested doses, 7.5 J/cm^2^ produced the most pronounced overall response. These findings support PBM as a promising adjunctive strategy for modulating endothelial behavior in vascular tissue engineering. By promoting a favorable balance between proliferation and cell survival, PBM may contribute to endothelialization-related processes relevant to this field.

## Supplementary Information

Below is the link to the electronic supplementary material.

Supplementary file1 (DOCX 16 KB)

## Data Availability

The datasets generated and/or analyzed during the current study are available from the corresponding author on reasonable request.
