# Supplementary material for: Photobiomodulation at 660 nm enhances proliferative activity while preserving viability in human endothelial cells in vitro
Source: Cell Tissue Bank. 2026 Jul 22;27(3):38. doi: 10.1007/s10561-026-10237-z (PMC13391784; doi:10.1007/s10561-026-10237-z)
Supplement: Supplementary file 1 — Supplementary file1 (DOCX 16 KB) [file 10561_2026_10237_MOESM1_ESM.docx]

**Supplementary Material**

**Supplementary Material 1. Calculation of Alamar Blue reagent reduction**The percentage reduction of Alamar Blue reagent was calculated using absorbance readings according to the manufacturer’s instructions as follows:

***% reduction of Alamar Blue reagent =*** $\frac{\left( \boldsymbol{E}\boldsymbol{oxi}\boldsymbol{600}\boldsymbol{\times}\boldsymbol{A}\boldsymbol{570} \right)\boldsymbol{-}\left( \boldsymbol{E}\boldsymbol{oxi}\boldsymbol{570}\boldsymbol{\times}\boldsymbol{A}\boldsymbol{600} \right)}{\left( \boldsymbol{E}\boldsymbol{red}\boldsymbol{570}\boldsymbol{\times}\boldsymbol{C}\boldsymbol{600} \right)\boldsymbol{-}\left( \boldsymbol{E}\boldsymbol{red}\boldsymbol{600}\boldsymbol{\times}\boldsymbol{C}\boldsymbol{570} \right)}$ ***× 100***

**where
Eoxi570** = the molar extinction coefficient of the oxidized Alamar Blue reagent at 570 nm = 80586
**Eoxi600** = the molar extinction coefficient of the oxidized Alamar Blue reagent at 600 nm = 117216
**A570** = the absorbance of the test wells at 570 nm
**A600** = the absorbance of the test wells at 600 nm
**Ered570** = the molar extinction coefficient of the reduced Alamar Blue reagent at 570 nm = 155677
**Ered600** = the molar extinction coefficient of the reduced Alamar Blue reagent at 600 nm = 14652
**C570** = the absorbance of the negative control well (medium + Alamar Blue reagent, no cells) at 570 nm
**C600** = the absorbance of the negative control well (medium + Alamar Blue reagent, no cells) at 600 nm.
